# Calcification Propensity of Serum is Independent of Excretory Renal Function

**DOI:** 10.1038/s41598-017-18336-4

**Published:** 2017-12-20

**Authors:** Bernhard Bielesz, Thomas Reiter, Rodrig Marculescu, Andreas Gleiss, Marija Bojic, Heidi Kieweg, Daniel Cejka

**Affiliations:** 1 Division of Nephrology and Dialysis, Department of Medicine III, Medical University of Vienna, Vienna, Austria; 2 Department of Laboratory Medicine, Medical University of Vienna, Vienna, Austria; 3 Center for Medical Statistics, Informatics, and Intelligent Systems, Medical University of Vienna, Vienna, Austria; 4 Department of Medicine III, Nephrology, Transplantation, Rheumatology, Geriatrics, Ordensklinikum Linz, Krankenhaus der Elisabethinen Linz, Linz, Austria

## Abstract

Vascular calcification is a component of cardiovascular disease, which is leading cause of death in patients with chronic kidney disease (CKD). A functional assay (T50-test) measuring the propensity of human serum to calcify associates with mortality and cardiovascular events in CKD patients. Calcification propensity is known to increase with CKD stage. We investigated whether the T50 readout is directly dependent on excretory kidney function (eGFR) or rather explained by deranged parameters of bone and mineral metabolism in the course of CKD. T50, along with markers implicated in calcification and mineral metabolism, were measured in a cross-sectional cohort of 118 patients with CKD stage 1–5. Associations of T50 with measured parameters were analysed and partial correlations performed to test to which extent the association of T50 with eGFR can be attributed to variation of these parameters. T50 correlates with eGFR, but serum levels of phosphate and calcium largely explain this association. Phosphate, magnesium, fetuin A, albumin, bicarbonate, and serum cross-laps but not Parathyroid Hormone or Fibroblast Growth Factor 23 are associated with T50 in multivariate adjusted models. These findings indicate that T50 values depend mainly on the concentration of promoters and inhibitors of calcification in serum, but not excretory kidney function.

## Introduction

Chronic kidney disease (CKD) is associated with increased all-cause and cardiovascular mortality^1^. Vascular calcification, which is part of a complex syndrome commonly referred to as chronic kidney disease – mineral bone disorder (CKD-MBD), is thought to be a major contributor to the excessively high cardiovascular risk in renal patients^2,3^. As renal function declines patients uniformly develop hyperphosphatemia, increased levels of parathyroid hormone (PTH) and fibroblast growth factor 23 (FGF23). All these factors have been associated with adverse clinical outcomes and increased mortality^4,5^.

Recently, a novel assay (T50-test) has been developed to measure the transformation time of amorphous to crystalline calciprotein particles at supersaturating conditions of calcium and phosphate^6^. T50 represents the time-point of half-maximal transformation of crystalline calciprotein particles (CPPs). Shorter T50 values in this assay are viewed as a reflection of increased calcification propensity of serum, whereas longer values indicate higher resistance of serum to calcification. Unlike merely measuring serum levels of parameters such as calcium, phosphate, PTH, and FGF23, T50 is a functional test that might better reflect the complex interplay of multiple components of the calcification defence system in serum.

A series of studies encompassing 5103 individuals has demonstrated associations of T50 with cardiovascular events, cardiovascular mortality, and overall mortality in patients with advanced CKD, dialysis patients, as well as renal transplant recipients^7–10^. Like phosphate, PTH, and FGF23, T50 associates with glomerular filtration rate thereby linking impaired calcification resistance with kidney function decline. However, it is currently unclear whether this association is already sufficiently explained by abnormalities in mineral metabolism (such as hyperphosphatemia), which are the consequence of CKD or rather mediated by reduced nephron mass along with a large number of known and unknown uremic toxins. The aim of the study was to test whether the association between renal function and propensity for calcification can be explained by the integrated action of known promoters and inhibitors of calcification which are deranged as a result of impaired kidney function. In a first step, we characterized the relationship of parameters involved in mineral and bone metabolism with T50 in a regression model adjusted for renal function. We further estimated the quantitative role of excretory renal function per se on calcification propensity in light of the observed alterations of mineral metabolism markers in progressive stages of chronic kidney disease.

## Results

118 patients with variable degrees of renal function impairment (estimated glomerular filtration rate (eGFR) range 113–6.6 ml/min/1.73 m^2^, median eGFR 37.8 ml/min/1.73 m^2^) were included in the study: diabetic nephropathy: 15; vascular nephropathy: 7; polycystic kidney disease: 11; glomerulonephritis: 27; interstitial nephritis: 1; other (HIV, tumour nephrectomy, systemic vasculitis, congenital ureteral disease and reflux, drug toxicity, cardiorenal - secondary due to heart failure, Alport syndrome): 28; undetermined aetiology: 29 (Table 1).

**Table 1:** Patient demographics.

	CKD I		CKD II		CKD IIIa		CKD IIIb		CKD IV		CKD V	
	mean/median	SD/IQR	mean/median	SD/IQR	mean/median	SD/IQR	mean/median	SD/IQR	mean/median	SD/IQR	mean/median	SD/IQR
n	16		22		14		23		24		19	
Sex (M/F)	10/6		8/14		8/6		17/6		14/10		9/10	
Age (years)	35.8	13.3	50	17	62.14	9.74	62.96	11.98	61.96	14.64	60.16	18.26
BMI	27.6	3.6	27.1	5.8	25.24	4.49	27.11	5.35	25.18	4.28	25.36	5.63
total calcium (mmol/l)	2.51	0.09	2.49	0.12	2.46	0.22	2.4	0.14	2.41	0.21	2.35	0.28
ionized calcium (mmol/l)	1.15	0.03	1.14	0.05	1.16	0.05	1.14	0.05	1.17	0.11	1.10	0.11
alb-corr calcium (mmol/l)	2.33	0.08	2.41	0.12	2.41	0.14	2.34	0.11	2.32	0.17	2.35	0.26
phosphate (mmol/l)	0.94	0.21	1.06	0.17	1.01	0.17	1.06	0.23	1.18	0.23	1.67	0.32
creatinine (mg/dl)	0.89	0.13	1.02	0.2	1.32	0.15	1.86	0.23	2.77	0.57	4.82	0.94
HCO_3_ (mmol/l)	25.47	1.57	25.13	1.82	23.29	3.3	23.43	2.05	21.5	2.01	21.75	3.48
protein (g/l)	73.4	3.3	71.1	6.41	72.14	6.15	71.03	5	72.87	6.04	66.95	4.73
albumin (g/l)	46.7	2.9	43.1	4.78	41.89	4.55	42.39	2.39	42.67	3.32	39.92	4.87
alkaline phosphatase (U/l)	61	54–76	61.5	51.25–79.5	67.5	52–95.75	81.5	64.5–124.3	72	52–84	77.5	57.25–110.3
CRP (mg/dl)	0.17	0.06–0.36	0.15	0.08–0.61	0.5	0.34–0.69	0.28	0.15–1.02	0.25	0.11–1.36	0.56	0.18–1.04
PTH (pg/ml)	22	18.5–26.3	35	26–68.5	41	29.5–63	63	46.25–104.3	103.5	69.5–142.8	134	53.25–396.5
CTX (ng/ml)	0.285	0.12–0.46	0.305	0.16–0.68	0.27	0.18–0.44	0.43	0.31	0.64	0.49–1.01	1.04	0.46–1.87
Osteocalcin (ng/ml)	19.9	15.1–27.7	16	10.85–39.8	18.4	11.35–25.85	35	18.18–50.45	42	28.28–92.4	98.9	32.1–198.3
P1NP (ng/ml)	50	41.3–75.5	42	23.5–78	36	22–49	58.5	33.75–103.3	74	54.75–145.5	120.5	68–327.5
magnesium (mmol/l)	0.78	0.09	0.78	0.07	0.78	0.11	0.81	0.09	0.82	0.18	0.77	0.1
protein/creatinine ratio (mg/g)	79.5	59.3–384.3	121	49.5–2380	89	26.3–1265	176	80.25–889.5	355	135.3–1269	1935	602.5–3153
sclerostin (pmol/l)	24.8	16.9–30.5	28	19.6–33.3	36.5	27.65–43.05	39.7	32.8–63.1	46.5	29.28–61.55	52.8	37.1–62.1
eGFR (ml/min/1.73 m²)	99.9	7.35	73.2	8.16	51.17	4.07	35.49	3.94	21.94	4.54	10.87	1.98
T50 (min)	288.8	54	268.8	54.68	260.8	51.24	248.6	59.74	238.9	51.65	191.9	65.86
cFGF23 (RU/ml)	70.7	53.1–124.6	110.6	77.63–163.3	162.9	102.6–198.7	265.4	187.2–525.1	379.1	297.7–592.1	1463	733.3–1804
iFGF23 (pg/ml)	58.12	55.3–62.56	68.19	51.02–79.08	96.47	78.44–110	112.3	99.7–191	155.7	122–247.1	547.2	254–1966
25(OH)D (nmol/l)	58.1	47.2–75.2	50.95	28.2–78.28	61.1	32.8–75.4	50.7	34–77.3	43.6	19.6–58.9	34.2	25.9–41.28
1,25(OH)_2_D (pmol/l)	171.6	130–218.4	144.1	118.3–176.8	91.8	79.9–167.7	100	84.2–137.6	78	60–110	50	40–90.9
Osteoprotegerin (pmol/l)	3.54	2.47–3.98	4.6	3.51–5.87	5.09	3.2–6.09	5.14	4.04–6.68	5.38	3.95–6.47	6.55	4.47–8.6
Fetuin A (ng/ml)	24.89	2.03	24.96	4.61	22.88	3.27	23.29	4.03	22.47	2.57	22.8	3.79
glu-Osteocalcin (ng/ml)	3.22	1.87–12.05	2.46	1.59–5.95	2.63	1.7–5.46	6.78	4.84–17.25	8.57	2.03–19.04	22.23	14.95–39.59

BMI: body mass index; alb-corr calcium: albumin-corrected calcium; PTH: parathyroid hormone; HCO_3_: serum bicarbonate; CTX: C-terminal telopeptide; P1NP: N-terminal propeptide of type 1 procollagen; eGFR: estimated glomerular filtration rate (CKD-EPI formula); T50: serum calcification propensity; cFGF23: c-terminal Fibroblast Growth Factor 23; iFGF23: intact Fibroblast Growth Factor 23; 25(OH)D: 25 hydroxy-vitamin D_3_; 1,25(OH)_2_D: 1, 25 dihydroxy-vitamin D_3_; glu-Osteocalcin: undercarboxylated Osteocalcin; CRP: C-reactive protein; shown are mean and standard deviation (±SD) for normally distributed or median and interquartile range (IQR) for skewed distributed data. Reference ranges: total calcium: 2.2–2.65 mmol/l; ionized calcium: 1.16–1.32 mmol/l; phosphate: 0.91–1.45 mmol/l; magnesium: 0.66–1.07 mmol/l; creatinine men: 0.70–1.20 mg/dl, women: 0.50–0.90 mg/dl; bicarbonate men: 24–31 mmol/l, women: 22–31 mmol/l; protein: 64–83 g/l; albumin: 35–52 g/l; alkaline phosphatase men: 40–130 U/l, women: 35–105 U/l; osteoprotegerin: (median) 2.7 pmol/l; PTH: 15–65 pg/mL; CTX men: 0.08–0.35 ng/ml, women: 0.09–0.44 ng/ml; osteocalcin men: 12–37 ng/ml (varies with age), women: 14–46 ng/ml (postmenopausal); P1NP men: 16–67 ng/ml (varies with age), women (postmenopausal): 28–92 ng/ml; 25(OH)D: 75–250 nmol/l; 1,25(OH)_2_D: 19.9–79.3 pg/ml; CRP: <0.5 mg/dl.

Serum Fetuin A, serum magnesium (Mg), phosphate, and intact Fibroblast Growth Factor 23 (iFGF23) showed a statistically significant influence on T50 after adjusting for age, gender, and eGFR (Table 2) with a proportion of T50 variation explained by age, gender, eGFR and the respective marker above 30% (R^2^ > 0.3). There was also a significant influence of serum bicarbonate (HCO_3_), albumin, protein, 1, 25 dihydroxy-vitamin D_3_ (1,25(OH)_2_D), and cross-laps (CTX), albeit with lower importance (R^2^ ≤ 0.3). No significant influence was found by total, ionized, and albumin-corrected calcium, PTH, c-terminal Fibroblast Growth Factor 23 (cFGF23), alkaline phosphatase, or other markers of bone turnover (Table 2). Based on well-established correlations of serum phosphate with FGF23, we further tested whether including phosphate in the adjusted model would alter our results^11^. After additional adjustment for phosphate, iFGF23 and 1,25(OH)_2_D were no longer significantly associated with T50, indicating that these relationships are mainly mediated by phosphate (Table S1).

**Table 2:** Crude and adjusted regression analysis of T50.

Marker	Unadjusted			Adjusted for age, gender, and eGFR			
	Beta	95% CI limits	p	Beta	95% CI limits	p	R^2^
Fetuin A (ng/ml)	11.3	8.7/13.8	**<0.001**	10	7.4/12.6	**<0.001**	0.53
serum magnesium (mmol/l)	164.7	66/263.4	**0.001**	166.3	80/252.5	**<0.001**	0.34
HCO_3_ (mmol/l)	8.5	4.3/12.7	**<0.001**	4.6	0/9.1	**0.049**	0.29
albumin (g/l)	5.5	2.8/8.1	**<0.001**	3.3	0.6/6	**0.017**	0.28
protein (g/l)	3.3	1.2/5.5	**0.002**	2.2	0.3/4.2	**0.027**	0.30
total calcium (mmol/l)	84.9	25.6/144.2	**0.005**	39.5	−16.7/95.7	0.166	0.26
ionized calcium (mmol/l)	167.8	10.1/325.6	**0.037**	108.8	−38.1/255.7	0.145	0.23
alb-corr calcium (mmol/l)	23.9	−50.1/97.9	0.523	0.8	−66.1/67.8	0.98	0.24
serum phosphate (mmol/l)	−113.8	−142.9/−84.8	**<0.001**	−108.2	−145/−71.4	**<0.001**	0.43
alkaline phosphatase* (U/l)	−23.8	−44.3/−3.4	**0.023**	−16.6	−35.2/1.9	0.078	0.26
CRP* (mg/dl)	−8	−14.1/−1.9	**0.010**	−3.1	−8.9/2.7	0.294	0.26
P1NP* (ng/ml)	−12.8	−22.9/−2.8	**0.013**	−4.6	−14.8/5.5	0.368	0.27
osteocalcin* (ng/ml)	−12.4	−21/−3.8	**0.005**	−1.7	−11.3/8	0.731	0.26
CTX* (ng/ml)	−18.9	−28/−9.8	**<0.001**	−11.2	−21.2/−1.2	**0.029**	0.29
PTH* (pg/ml)	−16.7	−25.3/−8	**<0.001**	−4.6	−14.6/5.5	0.368	0.27
cFGF23* (RU/ml)	−14.6	−21.4/−7.8	**<0.001**	−3	−13.1/7.1	0.561	0.26
iFGF23* (pg/ml)	−19.6	−26.2/−12.9	**<0.001**	−14.5	−24.4/−4.5	**0.005**	0.31
sclerostin* (pmol/l)	−24.5	−40.1/−8.9	**0.002**	7.6	−10.7/25.8	0.413	0.29
25(OH)D* (nmol/l)	17.9	3.2/32.6	**0.017**	9	−5/22.9	0.205	0.25
1,25(OH)_2_D* (pmol/l)	38.6	23.7/53.4	**<0.001**	21.5	2.6/40.4	**0.026**	0.28
glu-Osteocalcin* (ng/ml)	−11.1	−20.7/−1.5	**0.024**	−3.7	−14.3/6.9	0.488	0.25
osteoprotegerin* (pmol/l)	−15.6	−34.3/3	0.100	9	−11.2/29.1	0.38	0.25

Regression coefficients (beta) with T50 as dependent variable for respective markers from unadjusted linear regression models (left columns) and from regression models adjusting for gender, age, and eGFR (right columns). R^2^ values refer to adjustment variables plus the respective marker. ^*^ Indicates that calculations were performed with binary-log-transformed marker values such that beta quantifies the effect of doubling the marker; PTH: parathyroid hormone; HCO_3_: serum bicarbonate; CTX: C-terminal telopeptide; P1NP: N-terminal propeptide of type 1 procollagen; c-terminal Fibroblast Growth Factor 23; iFGF23: intact Fibroblast Growth Factor 23; 25(OH)D: 25 hydroxy-vitamin D_3_; 1,25(OH)_2_D: 1, 25 dihydroxy-vitamin D_3_; glu-Osteocalcin: undercarboxylated Osteocalcin; CRP: C-reactive protein; CI: confidence interval.

To quantify a putative independent role of renal function on T50 we performed unpartialized and partialized correlation analysis after classifying mineral and bone metabolism markers into the following groups based on pathophysiological considerations and known effects on T50 from previous data: class I (inhibitors of calcification): fetuin A, Mg, HCO_3_, Albumin; class II (promoters of calcification): calcium (total and ionized), phosphate; class III (anabolic bone markers): alkaline phosphatase, aminoterminal propeptide of type 1 procollagen, Osteocalcin; class IV (bone catabolism): CTX, PTH; class V (marker of nutritional state): serum protein; class VI (other outcome markers/inflammation): FGF23 (c-terminal and intact), Sclerostin, C-reactive protein (CRP). There was a weak but statistically significant correlation of T50 with eGFR in crude analysis (r = 0.41 (95% CI 0.24/0.56)). Assuming constant levels of phosphate alone largely blunted the correlation of T50 with eGFR (r: 0.15 (95% CI −0.04/0.33)) and slightly more so after adding calcium to the statistical analysis (class II: r = −0.02 (95% CI −0.23/0.18)). In contrast, other classes modified this association only in part (class I: r = 0.2 (95% CI 0/0.39), class III: r = 0.39 (95% CI 0.21/0.55), class IV: r = 0.32 (95% CI 0.13/0.48), class V: r = 0.39 (95% CI 0.22/0.54), class VI: r = 0.2 (95% CI 0.01/0.37)).

## Discussion

This is the first report that characterizes the relationship of a recently described serum calcification assay system with excretory kidney function and parameters of mineral metabolism and bone turnover in the full continuum of CKD stages I-V not on dialysis. It is a concise study outlining the relative importance of a fairly wide spectrum of mineral-metabolism parameters that influence a novel assay measuring calcification propensity in a functional manner. Our data set adds to previous reports on higher CKD stage populations and renal transplant recipients^7–10^.

Largely in agreement with previous findings, we observed significant dependence of T50 on Fetuin A, serum magnesium, serum phosphate, serum bicarbonate, serum albumin, serum protein, and 1,25(OH)_2_D^8,10^. We further found a significant association of iFGF23 but not cFGF23 with T50 in adjusted analysis. Although the reason for this discrepancy is unclear, distinct findings using both kinds of assays have been previously reported in healthy subjects^12^. However, both 1,25(OH)_2_D and iFGF23 correlate with serum phosphate (spearman: 1,25(OH)_2_D vs. phosphate r = −0.4; p < 0.001; phosphate vs. both iFGF23 and cFGF23 r = 0.5, p < 0.001). Further adjustment for serum phosphate blunted the associations of T50 with 1,25(OH)_2_D and iFGF23 suggesting that they are largely mediated by phosphate. In contrast, Fetuin A, HCO_3_, magnesium, protein, albumin, and CTX remained significant after adding phosphate to the regression model (Table S1). Interestingly, calcium (total, albumin-corrected or ionized) did not show robust associations with T50 in adjusted regression analysis.

The relatively weak but significant effect of bicarbonate on T50 might be explained by inherent changes of serum carbonate, which have been shown in earlier work to modify the transformation of amorphous calcium phosphate into crystalline apatite^13^. We further noted that among multiple hypothetical mediator classes the combination of phosphate and calcium (class II), but not PTH, FGF23, or bone metabolism markers, predominantly explain the increase of serum calcification propensity as renal function deteriorates.

The notion that eGFR no longer correlated with T50 after accounting for known promoters of calcification, namely phosphate and calcium, indicates that the myriad of known and unknown uremic toxins plays little residual role in this particular process. This is in line with recent data showing that live kidney donors do not demonstrate increases in calcification propensity despite a significant reduction in GFR after unilateral nephrectomy^14^. However, although higher calcium is assumed to promote calcification, the lack of a significant effect of calcium on T50 in multiple regression analysis and the ability of phosphate alone to blunt the correlation of T50 with eGFR suggest a predominant role of phosphate over calcium as determinant of T50.

Limitations are the cross-sectional study design, which precludes conclusions as to whether modifying one or more of the tested parameters would alter the T50 test result. An additional aspect pertains to the interpretation of Table 2 where higher beta values indicate larger changes in T50 after a one unit change along the respective scale (doubling for log-transformed data). Supplementing magnesium shows promise to improve T50, however, increasing serum levels by 1 mmol/l will not commonly be feasible for safety reasons. In contrast, lowering elevated phosphate levels in CKD V and haemodialysis patients by 1 mmol/l is a realistic objective and likely to improve T50 substantially. Future studies will need to address whether the combined optimization of the tested parameters according to T50-test results confers added benefit to the patient. Moreover, as the severity of vascular calcification is heterogeneous among CKD and also non-CKD patients such a test might also stratify patients at increased calcification risk early on.

We do acknowledge that the sizes of the CKD subgroups are not large enough to account for other potentially important factors such as the nature of the underlying kidney disease.

In conclusion, phosphate, magnesium, and fetuin A are major determinants of T50 in CKD I-V. Not excretory renal function itself but rather alterations in mineral homeostasis and serum protein composition, which are consequences of CKD, play a dominant role in determining calcification propensity.

## Methods

### Patients

The study protocol was approved by the ethics committee of the Medical University of Vienna and adhered to the Declaration of Helsinki. 118 chronic kidney disease patients stage 1 to 5 were recruited in the outpatient nephrology department after obtaining written informed consent. All eligible patients were invited to participate; exclusion criteria were age under 18, pregnancy, and history of kidney transplantation.

### Laboratory parameters

Calcification propensity (T50) was determined by Calciscon AG, Bern, Switzerland as described previously^6^. To achieve supersaturating conditions, calcium and phosphate were added to the serum samples to increase their final concentrations by 10 mM and 6 mM, respectively. Formation time of secondary, CPPs from primary, amorphous CPPs was measured by a nephelostar nephelometer (BMG Labtech, Ortenberg, Germany). Ionized calcium and serum bicarbonate were measured on routine blood gas analysis systems; total calcium, inorganic phosphate, magnesium, albumin, creatinine, and C-reactive protein were determined by routine assays (Cobas 8000, Roche Diagnostics, Germany) at the Department of Laboratory Medicine of the General Hospital of Vienna. Creatinine results were reduced by 5% to be traceable to isotope dilution mass spectroscopy (IDMS) values, which is the new reference method for creatinine measurements^15^. According to the respective manufacturer’s protocols the following parameters were determined by the indicated electrochemiluminescence immunoassays (ECLIA), chemiluminescence immunoassays (CLIA), radioimmunoassays (RIA) or enzyme-linked immunosorbent assays (ELISA): intact PTH, C-telopeptide of type I collagen for bone degradation, osteocalcin, and aminoterminal propeptide of type 1 procollagen ECLIA (Cobas 8000, Roche Diagnostics, Germany); undercarboxylated osteocalcin ELISA (MK118, Takara, South Korea); 25 hydroxy-vitamin D_3_ CLIA (Liaison, DiaSorin, Italy); 1, 25 dihydroxy-vitamin D_3_ solid phase extraction and RIA (DiaSorin, USA); intact Fibroblast Growth Factor 23 CLIA (DiaSorin, USA); Osteoprotegerin ELISA (BI-20403, Biomedica, Austria); Fetuin-A ELISA (RD191037100, BioVendor, Brno, Czech Republic); Sclerostin ELISA (BI-20492, Biomedica, Vienna, Austria); c-terminal Fibroblast Growth Factor 23 ELISA (60–6100, Immutopics, San Clemente, USA). All measurements were performed according to Good Laboratory Practice (GLP) standards.

### Statistics

Categorical variables are described by counts and percentages. Marker values are described by means and standard deviations if approximately normally distributed and by medians and interquartile ranges otherwise. The influence of markers on T50 is investigated by simple and multiple linear regression models; the latter are used to adjust for age, gender, (log-transformed) eGFR, and serum phosphate. Regression coefficients (beta) with 95% confidence intervals quantify the effect of a marker’s unit change on T50. Markers exhibiting a right-skewed distribution were transformed using binary logarithms such that beta refers to the effect of doubling the respective marker value. R^2^ measures for the multiple regression models quantify the markers’ relative importance (i.e. the proportion of variation in T50 explained by age, gender, eGFR, phosphate and the respective marker).

Crude and partial Spearman correlation coefficients (with 95% confidence intervals using Fisher’s z-transformation) are used to investigate to what extent the association between eGFR and T50 is reduced when classes of markers are kept constant.

No adjustment for testing multiple markers is done due to the exploratory character of this study. All calculations were done using SAS version 9.4 (SAS Institute, 2012).

### Data availability

The datasets generated during and/or analysed during the current study are available from the corresponding author on reasonable request.

## Electronic supplementary material

Table S1
