# Supplementary material for: Calcification Propensity of Serum is Independent of Excretory Renal Function
Source: Sci Rep. 2017 Dec 20;7:17941. doi: 10.1038/s41598-017-18336-4 (PMC5738386; doi:10.1038/s41598-017-18336-4)
Supplement: Supplementary file 1 — Table S1 [file 41598_2017_18336_MOESM1_ESM.pdf]

Calcification Propensity of Serum is Independent of Excretory Renal Function

Bernhard Bielesz, Thomas Reiter, Rodrig Marculescu, Andreas Gleiss, Marija Bojic, Heidi Kieweg, and Daniel Cejka

Table S1: Adjusted model from Table 2 including phosphate

	Adjusted for age, gender, eGFR, phosphate			
Marker	Beta	95% CI limits	p	R^2
Fetuin A (ng/ml)	10	8/12	**<0.001**	0.72
serum magnesium (mmol/l)	178.5	102.6/254.4	**<0.001**	0.52
HCO_3 (mmol/l)	4.5	0.5/8.5	**0.029**	0.46
albumin (g/l)	3.2	0.8/5.5	**0.009**	0.46
protein (g/l)	2.1	0.4/3.8	**0.016**	0.48
CTX* (ng/ml)	-9.6	-18.4/-0.9	**0.031**	0.46
cFGF23* (RU/ml)	1.4	-7.5/10.4	0.752	0.43
iFGF23* (pg/ml)	-4.1	-14/5.8	0.414	0.43
$1,25(OH)_2D$* (pmol/l)	14.2	-2.7/31.1	0.099	0.44

Legend Table S1:

Regression coefficients (beta) with T50 as dependent variable for respective markers from regression models adjusting for gender, age, eGFR, and phosphate. R^2 values refer to adjustment variables plus the respective marker. * indicates that calculations were performed with binary-log-transformed marker values such that beta quantifies the effect of doubling the marker; HCO_3: serum bicarbonate; CTX: C-terminal telopeptide; cFGF23: c-terminal Fibroblast Growth Factor 23; iFGF23: intact Fibroblast Growth Factor 23; $1,25(OH)_2D$: 1, 25 dihydroxy-vitamin D_3; CI: confidence interval
